# PCSK9 inhibitors for secondary prevention in patients with cardiovascular diseases: a bayesian network meta-analysis

**DOI:** 10.1186/s12933-022-01542-4

**Published:** 2022-06-15

**Authors:** Xing Wang, Dingke Wen, Yuqi Chen, Lu Ma, Chao You

**Affiliations:** 1 West China Hospital, Sichuan University, No. 37, Guo Xue Xiang, Chengdu, Sichuan 610041 People’s Republic of China; 2 West China Brain Research Centre, Sichuan University, Chengdu, Sichuan 610041 People’s Republic of China

**Keywords:** PCSK9 inhibitors, Secondary prevention, Cardiovascular disease, Atherosclerosis

## Abstract

**Background:**

The Food and Drug Administration has approved Proprotein Convertase Subtilisin/Kexin Type 9 (PCSK9) inhibitors for the treatment of dyslipidemia. However, evidence of the optimal PCSK9 agents targeting PCSK9 for secondary prevention in patients with high-risk of cardiovascular events is lacking. Therefore, this study was conducted to evaluate the benefit and safety of different types of PCSK9 inhibitors.

**Methods:**

Several databases including Cochrane Central, Ovid Medline, and Ovid Embase were searched from inception until March 30, 2022 without language restriction. Randomized controlled trials (RCTs) comparing administration of PCSK9 inhibitors with placebo or ezetimibe for secondary prevention of cardiovascular events in patients with statin-background therapy were identified. The primary efficacy outcome was all-cause mortality. The primary safety outcome was serious adverse events.

**Results:**

Overall, nine trials totaling 54,311 patients were identified. Three types of PCSK9 inhibitors were evaluated. The use of alirocumab was associated with reductions in all-cause mortality compared with control (RR 0.83, 95% CrI 0.72–0.95). Moreover, evolocumab was associated with increased all-cause mortality compared with alirocumab (RR 1.26, 95% CrI 1.04–1.52). We also found alirocumab was associated with decreased risk of serious adverse events (RR 0.94, 95% CrI 0.90–0.99).

**Conclusions:**

In consideration of the fact that both PCSK9 monoclonal antibody and inclisiran enable patients to achieve recommended LDL-C target, the findings in this meta-analysis suggest that alirocumab might provide the optimal benefits regarding all-cause mortality with relatively lower SAE risks, and evolocumab might provide the optimal benefits regarding myocardial infarction for secondary prevention in patients with high-risk of cardiovascular events. Further head-to-head trials with longer follow-up and high methodologic quality are warranted to help inform subsequent guidelines for the management of these patients.

**Supplementary Information:**

The online version contains supplementary material available at 10.1186/s12933-022-01542-4.

## Background

Patients who have had established cardiovascular diseases remain at elevated risks of recurrent cardiovascular events, leading to an increased risk of death [1, 2]. Therefore, secondary preventions targeting the established risk factors for this group of patients represent a high priority. For decades, statins have been regarded as the first-line drugs for lowering cholesterol levels and prevention of potential cardiovascular events. But a considerable proportion of high-risk hypercholesterolemic patients do not achieve adequate reductions in low-density lipoprotein cholesterol (LDL-C) despite of the intensive statin therapy [3]. According to the latest US and European guidelines, proprotein convertase subtilisin/kexin type 9 (PCSK9) inhibitors in combination with statin and ezetimibe therapy are recommended to reduce risk of cardiovascular events in these patients [2, 4].

PCSK9 accelerates degradation of LDL receptors, thereby inhibiting the removal of LDL from the circulation [5–7]. Thereafter, by controlling the expression of LDL receptor on the surface of hepatocytes, modulators that inhibit PCSK9 could reduce LDL-C and subsequently major cardiovascular events [8–10]. This therapy may be more effective in reducing LDL-C and other atherogenic lipids in high-risk patients treated with the maximum tolerated dose of statins, as well as those who are intolerant to statins. Although there are safety concerns such as the potential risk of new-onset diabetes [11–13], several meta-analyses have demonstrated that PCSK9 inhibitors showed better effects in reducing LDL-C levels and improving clinical benefits than other lipid-lowering agents for the secondary prevention of cardiovascular disease [14, 15]. However, due to the lack of direct comparisons between different medications, the optimal agent targeting PCSK9 to reduce the risk of death after cardiovascular events remains undetermined. Therefore, this study aimed at evaluating the efficacy and safety of different PCSK9 inhibitors for secondary prevention in patients with high-risk of cardiovascular events.

## Methods

### Guidance and protocol

The methodology for reporting the systematic review with network meta-analysis followed the PRISMA-NMA guideline [16]. The protocol of the present study was registered in Open Science Framework database (https://osf.io/xf9dh).

### Data sources and search strategy

Several electronic databases were searched, including Ovid Medline, Ovid Embase, and Cochrane Library of Clinical Trials. Searches were conducted from inception until March 30, 2022, without restrictions of language or publication status. The following MesH terms and their entry terms were chosen: “PCSK9 Inhibitors”, “hypercholesterolemia”, “randomized controlled trial”. For any ongoing studies or completed studies with reported results, we consulted the relevant clinical trials registry (https://www.clinicaltrials.gov/). We also inspected the reference lists of included trials and latest reviews in the same field. The details of the search strategy conducted are presented in Additional file 1: Table S1.

### Selection criteria

We only included randomized controlled trials that met the following criteria: first, the study population should be adult patients (age ≥ 18) with established coronary heart disease (CHD), atherosclerotic cardiovascular disease (ASCVD), or disease risk equivalent; second, the intervention group used PCSK9 modulating therapies for secondary prevention with statin background therapy; third, comparison group was placebo or ezetimibe, or a different PCSK9 modulating therapy; forth, at least one outcome of the following had to be reported. The primary efficacy outcome was all-cause mortality, and the primary safety outcome was serious adverse events (SAEs). Follow-up duration of the cardiovascular events should be at least 48 weeks or one year. Secondary efficacy outcomes including cardiovascular death, myocardial infarction, and stroke. Secondary safety outcomes including injection site reaction, new-onset diabetes, and neurocognitive disorders. These outcomes could be defined by each trial.

### Study selection and data extraction process

Study selection was carried out by two authors (XW and DW) independently. Most of the literature was excluded based on the titles and abstracts of all publications retrieved in the electronic search. Only when both agreed that literature met the eligibility criteria did they screen the full text for potentially relevant trials. In cases of any disagreements, the problems were resolved by detailed discussion between the study team. When inclusion criteria needed to be assessed or vital data were missing, corresponding authors were responsible for contacting to obtain the missing information.

Data extraction and collection process were performed by two independent authors (XW and DW) using predesigned table forms. Any disagreements were resolved by detailed discussion between the study team.

### Quality assessment

Risk of bias assessments of the eligible studies were completed by two authors (XW and LM) independently using the Cochrane risk of bias assessment tool [17]. For each study, the following six domains needed to be assessed: first, selection bias including allocation sequence concealment and random sequence generation, second, detection bias including blinding of outcome assessment, third, performance bias including blinding of participants and personnel, forth, reporting bias including selective reporting, fifth, attribution bias including incomplete of outcome data, and sixth, other potential sources of bias.

Assessments of certainty of evidence were performed by two authors (XW and CY) using the Grading of Recommendations, Assessment, Development and Evaluation (GRADE) designed by the GRADE working group. The following five aspects need to be taken into account: first, overall risk of bias, second, imprecision, third, inconsistency, forth, publication bias, and fifth, indirectness [18].

### Data synthesis and analysis

The statistical analyses were performed using the R packages in R software (version 4.0.5) and RevMan (version 5.4.0). We performed Bayesian network meta-analyses using a consistency model to incorporate indirect comparisons. In brief, the comparison of the effect of any two treatment regimens as a function was modeled that each drug was relative to the reference drug. Dichotomous variables were expressed as risk ratios (RR), and continuous variables were expressed as mean difference (MD). The corresponding 95% credible interval (CrI) was obtained using the 2.5th and 97.5th percentiles of the posterior distribution. The models are based on 30,000 iterations after a burn-in of 10,000 iterations. For the inadequate convergence of the model, the parameters were further modified until a satisfactory convergence was achieved. The rankograms were estimated to rank the intervention hierarchy in the network meta-analysis. We used the surface under the cumulative ranking curve (SUCRA) to estimate the ranking probability of the treatment agents for each outcome. Heterogeneity of the model was assessed using with the Chi^2^ test and the I^2^ test. A I^2^ vlaue of more than 50% was considered substantial [19]. Tests of statistical significance were based on two side and a p value with less than 0.05 was considered as statistically significant. The possibility of publication bias was evaluated by the Harbord regression test, Egger regression test, and Begg’s test if more than ten trials were included [20].

## Results

### Study selection and characteristics

Through a systematic database search, we identified 1,478 records. After selection, nine trials totaling 54,311 participants fulfilled the aforementioned criteria and were included in the analysis [21–28]. The study selection process was presented in the Additional file 1: Figure S1 in the Supplement. Characteristics of the eligible trials are presented in Table 1. Five trials compared alirocumab with control, two trials compared evolocumab with control, and two trials compared inclisiran with control. Study sizes ranged from 300 to 27,564 participants; the mean age ranged from 58.6 to 65.7 years; the percentage of male participants ranged from 63.3% to 81.0%.

**Table 1:** Characteristics of studies included in the systematic review

Trial	Register number	Patients, n	Male (%)	Age, years*	BMI, kg/m^2^*	Diabetes (%)*	Follow up (weeks)	Baseline for LDL-C (mg/dl)*	Characteristics of patients	Background therapy	Administration of PCSK9 modulators
PACMAN-AMI 2022	NCT03067844	300	81.0	58.6	28.2	12.5	52 weeks	150.9	Adults with STEMI or NSTEMI	High-intensity statin therapy	Alirocumab 150 mg every 2 weeks
ODYSSEY OUTCOMES 2018	NCT01663402	18,924	74.8	58.6	28.5	29.1	2.8 years	92	Adults with an acute coronary syndrome 1 to 12 months before randomization	Maximally tolerated statin therapy	Alirocumab 75 mg every 2 weeks
ODYSSEY COMBO I 2015	NCT01644175	316	65.8	63.0	32.0	39.3	52 weeks	106.0	Adults with established CHD or CHD risk equivalents	Maximally tolerated statin therapy	Alirocumab 75 mg every 2 weeks
ODYSSEY COMBO II 2015	NCT01644188	720	73.6	61.3	30.3	31.5	52 weeks	104.4	Adults with established CHD or CHD risk equivalents	Maximally tolerated statin therapy	Alirocumab 75 mg every 2 weeks
ODYSSEY LONG TERM 2015	NCT01507831	2,341	63.3	60.6	30.5	33.9	78 weeks	121.9	Adult patients with heterozygous familial hypercholesterolemia or with established CHD or a CHD risk equivalent	Maximally tolerated statin therapy	Alirocumab 150 mg every 2 weeks
FOURIER 2017	NCT01764633	27,564	75.4	62.5	NR	36.5	2.2 years	92	Adults with clinically evident ASCVD	High-intensity or moderate-intensity statin therapy	Evolocumab 140 mg every 2 weeks or 420 mg every month
GLAGOV 2016	NCT01813422	968	72.2	59.8	29.5	21.5	78 weeks	92.4	Adults with angiographic coronary disease	High-intensity or moderate-intensity statin therapy	Evolocumab 420 mg every month
ORION- 10 2020	NCT03399370	1,561	69.3	65.7	NR	42.4	540 days	104.8	Adults with ASCVD	Maximally tolerated statin therapy	Inclisiran 284 mg day 1, day 90, and every 6 months thereafter
ORION- 11 2020	NCT03400800	1,617	71.7	64.8	NR	33.7	540 days	103.7	Adults with ASCVD or an ASCVD risk equivalent	Maximally tolerated statin therapy	Inclisiran 284 mg day 1, day 90, and every 6 months thereafter

*NR* not reported, *BMI* body-mass index, *LDL-C* low-density lipoprotein cholesterol, *CHD* coronary heart disease, *ASCVD* atherosclerotic cardiovascular disease, *STEMI* ST-elevation myocardial infarction, *NSTEMI*: non-ST-elevation myocardial infarction

^*^from control group

### Efficacy outcomes

**Fig. 1:**
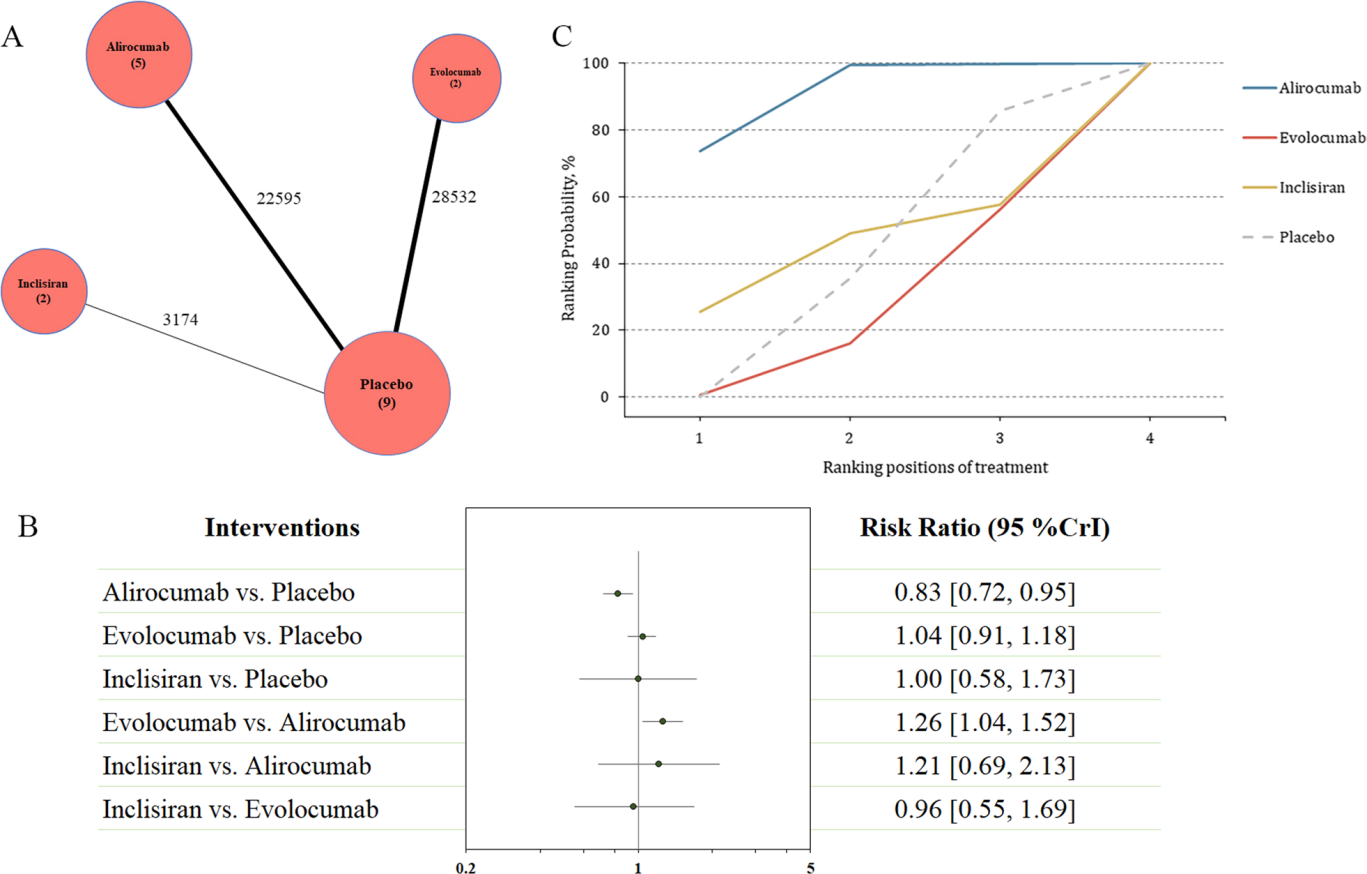
Summary of the primary efficacy outcome. **A** Network plot of all-cause mortality. The width of the lines is proportional to the number of studies comparing every pair of treatments, and the size of each circle is proportional to the number of participants. **B** The forest plot shows the risk ratio (RR) and credible interval (CrI). **C** SUCRA-based ranking probabilities graph of each medication. The SUCRA values for each treatment were as follows: 91% for alirocumab; 24% for evolocumab; 44% for inclisiran. *SUCRA* surface under the cumulative ranking curve

**Fig. 2:**
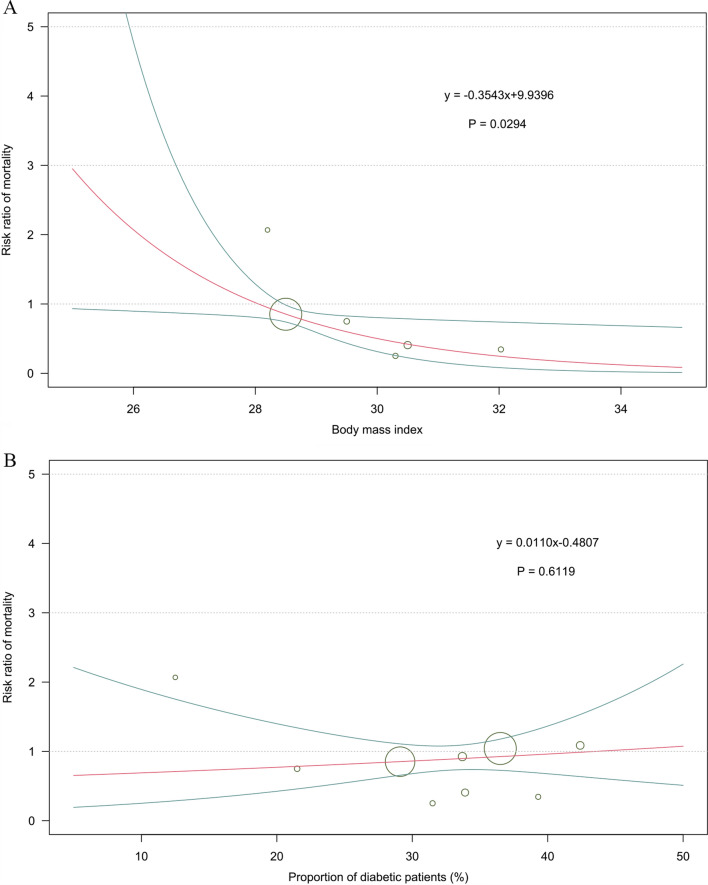
**A** Meta-regression analysis for the interaction of BMI on the risk of all-cause mortality. The BMI value was extracted from the control group in each trial. *BMI* body mass index. **B** Meta-regression analysis for the interaction of proportion of diabetic patients on the risk of all-cause mortality. The diabetes data was extracted from the control group in each trial

All the included studies reported the primary efficacy outcome, including a total of 54,301 participants with available data in terms of the all-cause mortality (Fig. 1A). The administration of alirocumab was associated with reductions in all-cause mortality compared with control (RR 0.83, 95% CI 0.72–0.95; Fig. 1B). Evolocumab was associated with increased all-cause mortality compared with alirocumab (RR 1.26, 95% CI 1.04–1.52; Fig. 1B). The SUCRA value represents the overall rank for each agent with regards to the likelihood of the outcome of interest (Fig. 1C). Alirocumab was identified as the best regimen that results in reduction of all-cause mortality, with a SUCRA value of 0.91. This result also showed significant difference. Followed treatment agents were inclisiran (SUCRA = 0.44), and evolocumab (SUCRA = 0.24). We performed sensitivity analysis by excluding the ODYSSEY LONG TERM trial which enrolled some patients with heterozygous familial hypercholesterolemia (< 20%), and the results remained consistent (Table 2). Meta-regression was performed to test the effects of body mass index (BMI) and diabetes on the risk of death. The results were shown in Fig. 2, which revealed a negative interaction between the BMI and the death risk (p = 0.029; Fig. 2).

**Table 2:** Sensitivity analysis by excluding the ODYSSEY LONG TERM trial

Intervention	All-cause mortality	Serious adverse events
	RR (95% CrI)	RR (95% CrI)
Compared with placebo		
Alirocumab	0.84 [0.73, 0.97]	0.94 [0.90, 0.99]
Evolocumab	1.04 [0.91, 1.18]	1.00 [0.96, 1.04]
Inclisiran	1.00 [0.58, 1.72]	0.92 [0.81, 1.04]
Compared with alirocumab		
Evolocumab	1.24 [1.02, 1.50]	1.06 [1.00, 1.13]
Inclisiran	1.18 [0.67, 2.08]	0.98 [0.85, 1.12]
Compared with evolocumab		
Inclisiran	0.96 [0.55, 1.68]	0.92 [0.80, 1.05]

*RR* relative risk, *CrI* credibility interval

**Fig. 3:**
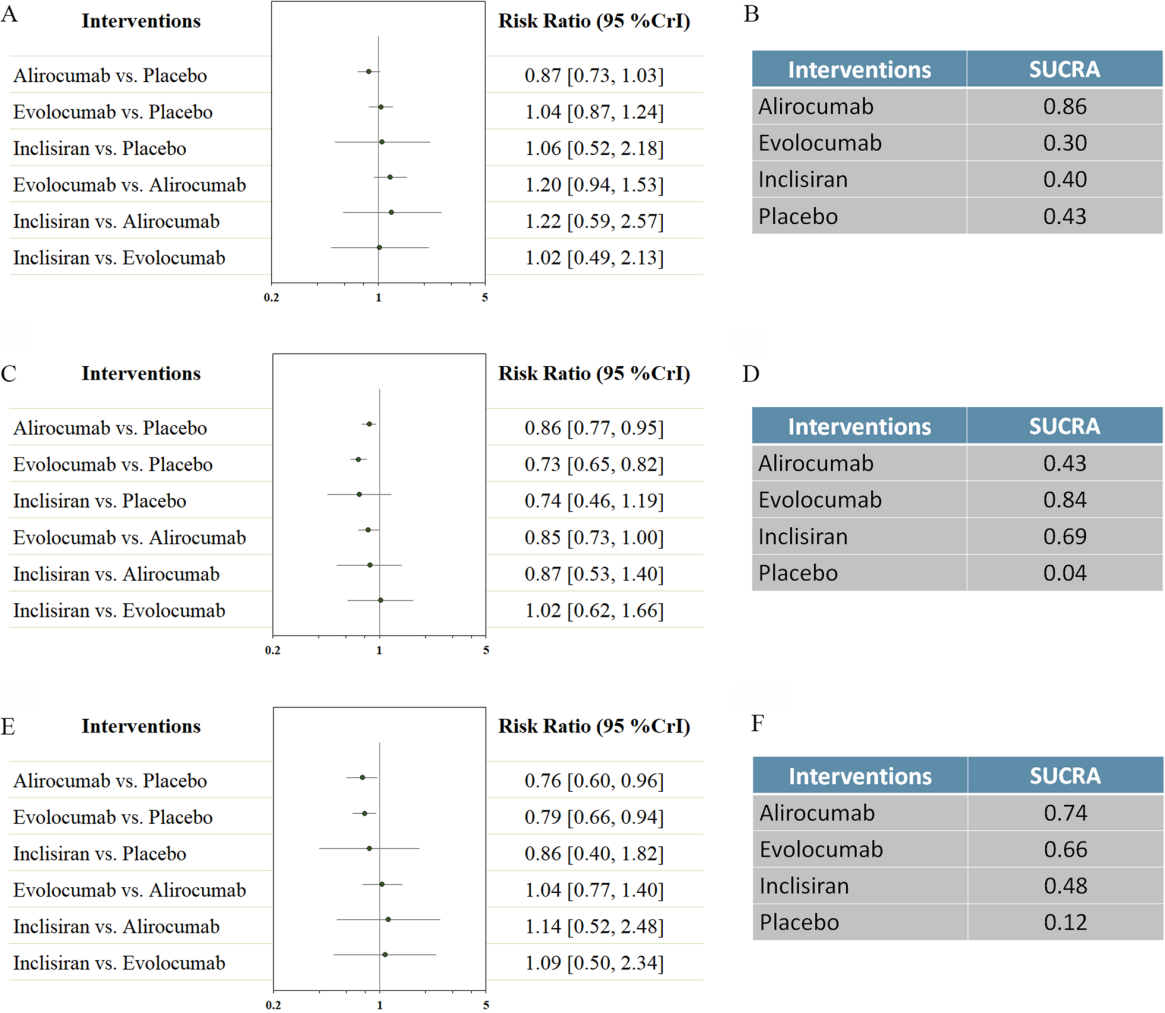
Network analysis for secondary efficacy outcomes. **A** The forest plot for cardiovascular death. **B** The SUCRA value of each treatment for cardiovascular death. **C** The forest plot for myocardial infarction. **D** The SUCRA value of each treatment for myocardial infarction. **E** The forest plot for stroke. **F** The SUCRA value of each treatment for stroke. *CrI* credible interval, *SUCRA* surface under the cumulative ranking curve

Other cardiovascular events were reported in Fig. 3. No difference was found in cardiovascular death. Alirocumab was ranked the most efficacious in reducing cardiovascular death (SUCRA = 0.86). Both alirocumab and evolocumab were associated with reductions in myocardial infarction (RR 0.86, 95% CI 0.77–0.95; and RR 0.73, 95% CI 0.65–0.82 respectively), and stroke (RR 0.76, 95% CI 0.60–0.96; and RR 0.79, 95% CI 0.66–0.94 respectively). Evolocumab was ranked the most efficacious in reduction of myocardial infarction (SUCRA = 0.84), while alirocumab was the most effective treatment in reducing risk of stroke (SUCRA = 0.74).

### Safety outcomes

**Fig. 4:**
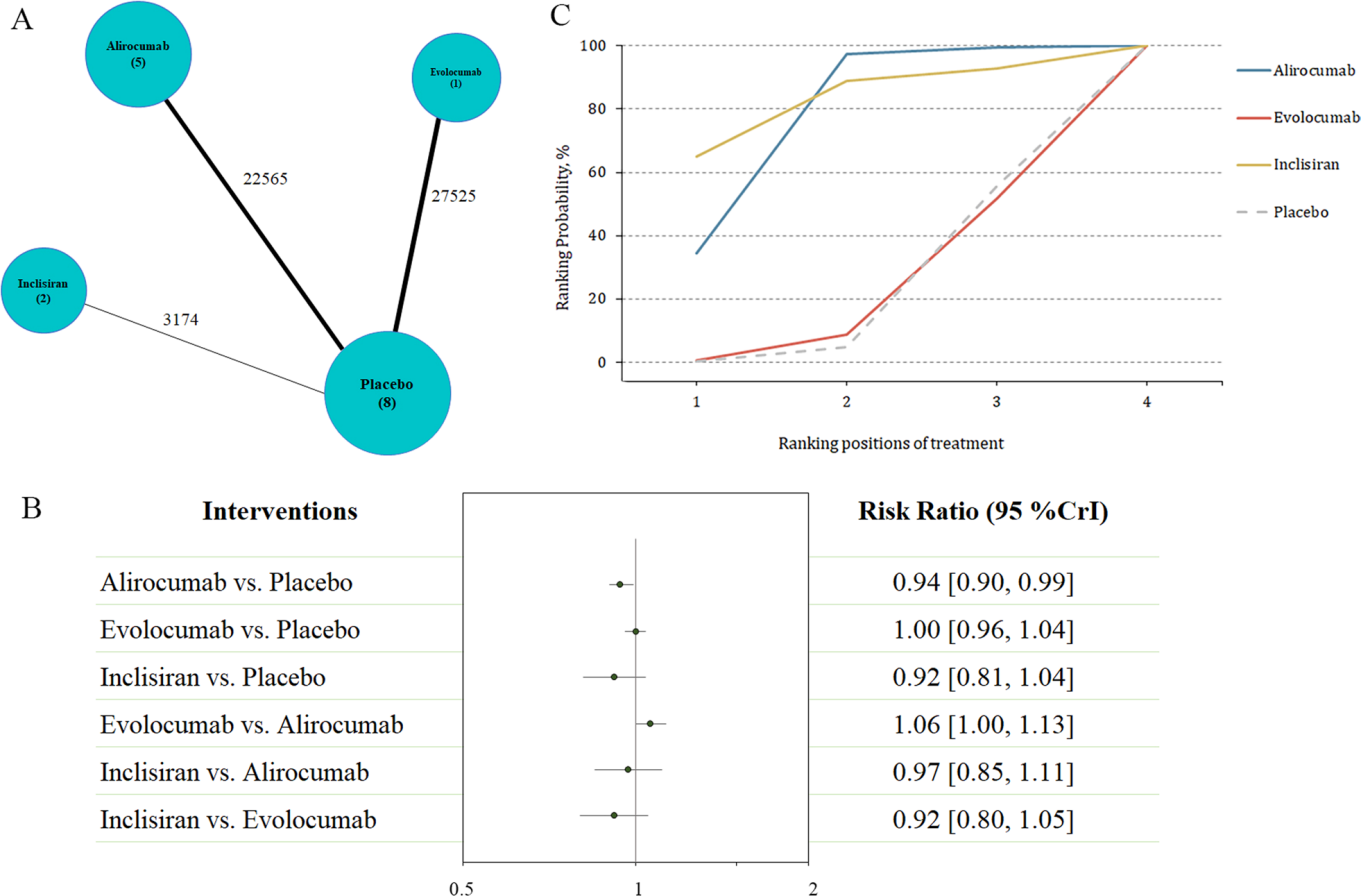
Summary of the primary safety outcome. **A** Network plot of serious adverse events. The width of the lines is proportional to the number of studies comparing every pair of treatments, and the size of each circle is proportional to the number of participants. **B** The forest plot shows the risk ratio (RR) and credible interval (CrI). **C** SUCRA-based ranking probabilities graph of each medication. The SUCRA values for each treatment were as follows: 77% for alirocumab; 20% for evolocumab; 83% for inclisiran. *SUCRA* surface under the cumulative ranking curve

A total of eight trials reported serious adverse events, including 53,264 patients (Fig. 4A). The administration of alirocumab was associated with reductions in serious adverse events compared with control (RR 0.94, 95% CI 0.90–0.99; Fig. 4B). SUCRA curve identified inclisiran (SUCRA = 0.83; Fig. 4C) as the top ranked treatment in association with less serious adverse events, followed by alirocumab (SUCRA = 0.77; this result was also significant) and evolocumab (SUCRA = 0.20). Similar results were obtained for the primary safety outcome by excluding the ODYSSEY LONG TERM trial (Table 2).

**Fig. 5:**
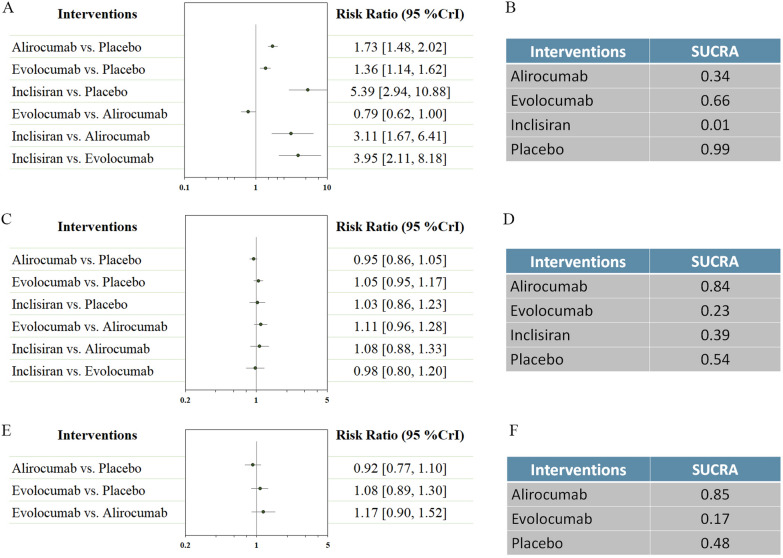
Network analysis for secondary safety outcomes. **A** The forest plot for injection site reaction. **B** The SUCRA value of each treatment for injection site reaction. **C** The forest plot for new-onset diabetes. **D** The SUCRA value of each treatment for new-onset diabetes. **E** The forest plot for neurocognitive disorders. **F** The SUCRA value of each treatment for neurocognitive disorders. *CrI* credible interval, *SUCRA* surface under the cumulative ranking curve

**Fig. 6:**
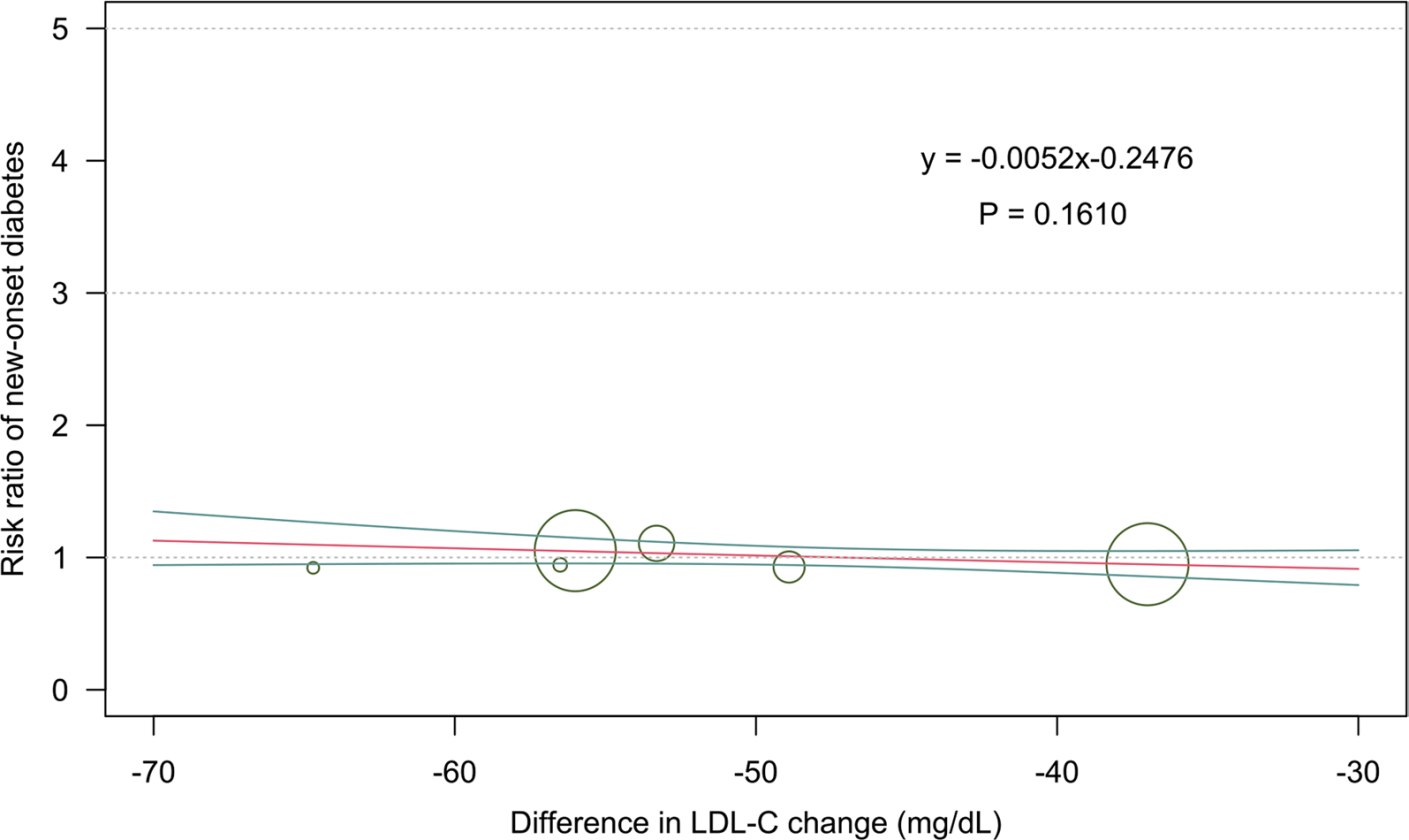
Meta-regression analysis for the interaction of difference between PCSK9 inhibitors group and control group in LDL-C level change on the risk of new-onset diabetes. *LDL-C* low-density lipoprotein cholesterol, *PCSK9* proprotein convertase subtilisin/kexin 9

Other safety outcomes were reported in Fig. 5. The use of alirocumab, evolocumab, and inclisiran were associated with increased risk in injection site reaction (RR 1.73, 95% CI 1.48–2.02; RR 1.36, 95% CI 1.14–1.62; and RR 5.39, 95% CI 2.94–10.88 respectively). Therapies with alirocumab, evolocumab, and inclisiran were not associated with an increased incidence of new-onset diabetes, and neurocognitive disorders. Evolocumab ranked the best strategy for injection site reaction (SUCRA = 0.66), while alirocumab as the best agent for new-onset diabetes (SUCRA = 0.84), and neurocognitive disorders (SUCRA = 0.85). We also evaluated the effects of LDL-C change on the risk of new-onset diabetes (Fig. 6). The results did not show any significant interactions (p = 0.161).

### Quality assessments

The overall quality of the nine included trials was judged to be high (Additional file 1: Figures S2 and S3). The certainty of the evidence for the network comparisons of alirocumab vs. placebo in the primary efficacy outcome was judged as high; alirocumab vs. evolocumab in in the primary efficacy outcome was judged as low due to indirectness and imprecision. The quality of the evidence for the network comparisons of alirocumab vs. placebo in the primary safety outcome was judged as high; alirocumab vs. evolocumab in in the primary safety outcome was judged to be low due to indirectness and imprecision.

## Discussion

Cardiovascular disease is one of the leading causes of death, accounting for approximately one third of deaths in the United States [29]. In the present meta-analysis of nine RCTs totaling 54,311 patients, we evaluated the comparative effect of three PCSK9 inhibitors in the secondary prevention in patients with high-risk of ASCVD. We excluded trials that compared bococizumab with placebo because it was dumped in 2016 by its manufacture. Reasons for withdrawal included unexpected attenuation of LDL-C-lowering effects over time, and higher rates of immunogenicity and injection site reactions during treatment than with other drugs in this class [30, 31]. According to the present analysis, the use of alirocumab was associated with reductions in the all-cause mortality and serious adverse event. Besides, administration of evolocumab was associated with decreased risk of myocardial infarction.

### Comparison with the latest evidences

This study is the first network meta-analysis assessing the effect of different modulators targeting PCSK9 on cardiovascular events in patients with ASCVD to the best of our knowledge. Previous studies have been performed to assess the comparative effects of PCSK9 inhibitors, statins, and ezetimibe. The authors concluded that PCSK9 inhibitors were ranked as the most effective treatment for reducing cardiovascular events without increasing major safety concerns [14]. Thus, it is important to explore the optimal PCSK9 inhibitors which benefit high-risk patients the most. Former meta-analyses have evaluated the effects of different PCSK9 modulators compared to controls through direct comparisons. Most of them did not find significant differences regarding all-cause mortality [15, 32–34]. Our study found that evolocumab significantly reduced the risk of myocardial infarction. Similar findings have been found in other studies [15, 35, 36]. Moreover, a recent study demonstrated that the combination of evolocumab and statin produced favorable changes in coronary atherosclerosis after non-ST-segment elevation myocardial infarction, consistent with stabilization or even regression [37].

Most of the previous studies on the same field were designed as direct meta-analyses, which provided only partial information in this case and therefore did not optimally inform decision making on comparative effectiveness of different treatment agents. The present study used network analysis which could help evaluate comparative effectiveness of various treatment agents [35, 38] This method is useful to improve the precision of the outcome estimate and allows estimation of the comparative effectiveness of different types of PCSK9 inhibitors.

Another notable finding from the meta-regression was that risk of all-cause mortality was statistically significantly lower in patients with higher BMI. This finding suggest that these patients might be more likely to benefit from treatment with monoclonal antibodies targeting PCSK9. On the other hand, recent studies demonstrated that loss-of-function variants in PCSK9 were associated with lower LDL-C levels but associated with increased levels of fasting glucose concentration and an increased risk for new-onset diabetes, which resulted in serious concerns about the safety of the anti-PCSK9 treatments [11–13]. According to our analysis, there is no significant impact of LDL-C change induced by PCSK9 inhibitors on new-onset diabetes.

### Mechanism and clinical implications

PCSK9 binds to LDL receptors on the hepatocytes surface and induces degradation of them after internalization, resulting in reduced uptake of LDL-C by the liver and increased levels of circulating LDL-C. PCSK9 inhibitors exert lipid-lowering effects by decreasing plasma PCSK9, ultimately leading to a reduction in the major cardiovascular events [39]. These agents could not only effectively decrease levels of LDL-C, but also reduce apolipoprotein B (apoB), lipoprotein (a) [Lp(a)], and non-HDL-C levels. The lipid-lowering potential in addition to LDL-C was observed both in PCSK9 monoclonal antibody and inclisiran [40]. Furthermore, it has been reported that more individuals with type 2 diabetes mellitus (T2DM), with and without atherogenic dyslipidemia, achieve the recommended LDL-C targets compared to those without T2DM [41, 42].

Although both PCSK9 monoclonal antibody and inclisiran upregulate LDL receptors and thereby reduce LDL-C concentrations by diminishing active PCSK9, their mechanisms of action are different. Monoclonal antibodies function extracellularly to bind and block circulating PCSK9 protein, still allowing PCSK9 to be produced intracellularly [43]. Inclisiran works intracellularly by preventing the translation of PCSK9 mRNA, thereby decreasing both intracellular and plasma PCSK9 levels [44]. A potential advantage of treatment with inclisiran is the longer duration of its lipid-lowering effect. As a result, the frequency of administration is less compared to PCSK9 mAbs. Specifically, inclisiran required subcutaneous injections once every six months, whereas PCSK9 mAbs should be injected once every 2–4 weeks. Different administration patterns may lead to differences in the development of adverse events, particularly injection site reactions, which should be taken into account when choosing the appropriate agent [7, 45].

Recently, a rapid recommendation is published, showing a clinical practice guideline of PCSK9 inhibitors for the reduction of cardiovascular events in patients at different risks [46]. The guideline panel provided weak recommendations to add a PCSK9 inhibitor to ezetimibe for adults already taking statins at very high risk of cardiovascular event and those at very high and high risk who are intolerant to statins. In consideration of the fact that both PCSK9 monoclonal antibody and inclisiran enable patients to achieve recommended LDL-C target. Our study revealed that the use of alirocumab was associated with reductions in the all-cause mortality and serious adverse event. Besides, administration of evolocumab was associated with decreased risk of myocardial infarction. The findings of this study update current guidelines in a novel way.

## Strengths and limitations

Given limited comparative effectiveness of different types of PCSK9 inhibitors for secondary prevention in patients with high-risk of cardiovascular events, a Bayesian network meta-analysis was established. To determine the best approach benefiting the patients most, we used all-cause mortality within at least one year follow up to evaluate the efficacy, and serious adverse events to evaluate the safety. Besides, we followed the guidelines of the PRISMA-NMA statement; included explicit eligibility criteria; and performed a comprehensive search strategy. We also included GRADE to assess certainty in pooled estimates of effect and presented absolute and relative risks. Thus, our analysis is robust and extending and integrating the recent guidelines in a novel way.

This study has several limitations. First, in some of the comparisons, we did find a significant difference. For example, alirocumab showed better efficacy in reducing all-cause mortality than evolocumab, and evolocumab was superior to alirocumab in reducing risk of myocardial infarction. However, the result of these outcomes might be imprecise and heterogeneous because direct head-to-head studies were lacked. We have downgraded the quality of evidence of these outcomes.

Second, clinical heterogeneity existed regarding the dosage and administration interval among the different treatment regimens. For example, PCSK9 monoclonal antibody needs to be administered 1–2 times per month, while inclisiran can be given only once every 6 months. Clinicians need to make comprehensive considerations in selecting the appropriate agents based on administration intervals, effects, and cost-effectiveness [47, 48].

Third, in the present study, the maximum follow-up period of the included trials was 2.8 years. More trials with longer follow-up are required to examine whether the benefits of PCSK9 inhibitors will emerge over time.

## Future research

The findings of the present analysis suggest that more clinical trials are needed to investigate the efficacy and safety of different types of PCSK9 inhibitors on cardiovascular outcomes. We searched the National database of clinical trials (https://www.clinicaltrials.gov/) to identify any ongoing trials. A Phase III clinical trial (NCT04790513) is currently in progress to evaluate the efficacy and safety of LIB003, evolocumab, and alirocumab in patients with cardiovascular disease. In addition, it is thought that PCSK9 inhibition provides a definite cardiovascular benefit by lowering LDL-C levels, but may increase the risk of new-onset diabetes [7]. Longer follow-ups could be helpful to provide much more information on effectiveness, long-term safety, and tolerability of PCSK9 inhibitors.

## Conclusions

In consideration of the fact that both PCSK9 monoclonal antibody and inclisiran enable patients to achieve recommended LDL-C target, the findings in this meta-analysis suggest that alirocumab might provide the optimal benefits regarding all-cause mortality with relatively lower SAE risks, and evolocumab might provide the optimal benefits regarding myocardial infarction for secondary prevention in patients with high-risk of cardiovascular events. In the absence of multi-arm RCTs that include treatment regimens with various agents targeting PCSK9, our exploration provides an important and useful guide to inform treatment decisions. Further head-to-head trials with longer follow-up and high methodologic quality are warranted to help inform subsequent guidelines for the management of these patients.

## Supplementary Information

**Additional file 1: Table S1**. Search Strategy. **Figure S1**. Study selection flowchart of randomized controlled trials. **Figure S2**. Risk of bias summary. **Figure S3**. Risk of bias graph.

## Data Availability

All data generated or analyzed during this study are included in this published article and in its Additional file.

## Abbreviations

ASCVD: Atherosclerotic cardiovascular disease
BMI: Body Mass Index
CHD: Coronary heart disease
CrI: Credible interval
GRADE: Recommendations, Assessment, Development and Evaluation
LDL-C: Low-density lipoprotein cholesterol
PCSK9: Proprotein Convertase Subtilisin/Kexin Type 9
RR: Risk ratio
SUCRA: Surface under the cumulative ranking curve
T2DM: Type 2 diabetes mellitus

## References

[1] Dhamoon MS, Sciacca RR, Rundek T, Sacco RL, Elkind MS (2006). Recurrent stroke and cardiac risks after first ischemic stroke: the Northern Manhattan Study. Neurology.

[2] Mach F, Baigent C, Catapano AL, Koskinas KC, Casula M, Badimon L (2020). 2019 ESC/EAS Guidelines for the management of dyslipidaemias: lipid modification to reduce cardiovascular risk. Eur Heart J.

[3] Mora S, Wenger NK, Demicco DA, Breazna A, Boekholdt SM, Arsenault BJ (2012). Determinants of residual risk in secondary prevention patients treated with high- versus low-dose statin therapy: the Treating to New Targets (TNT) study. Circulation.

[4] Wilson PWF, Polonsky TS, Miedema MD, Khera A, Kosinski AS, Kuvin JT (2019). Systematic Review for the 2018 AHA/ACC/AACVPR/AAPA/ABC/ACPM/ADA/AGS/APhA/ASPC/NLA/PCNa guideline on the management of blood cholesterol: a report of the American College of Cardiology/American Heart Association Task Force on Clinical Practice Guidelines. J Am Coll Cardiol.

[5] Lin XL, Xiao LL, Tang ZH, Jiang ZS, Liu MH (2018). Role of PCSK9 in lipid metabolism and atherosclerosis. Biomed Pharmacother.

[6] Cameron J, Bogsrud MP, Tveten K, Strøm TB, Holven K, Berge KE (2012). Serum levels of proprotein convertase subtilisin/kexin type 9 in subjects with familial hypercholesterolemia indicate that proprotein convertase subtilisin/kexin type 9 is cleared from plasma by low-density lipoprotein receptor-independent pathways. Transl Res.

[7] Macchi C, Ferri N, Sirtori CR, Corsini A, Banach M, Ruscica M (2021). Proprotein convertase subtilisin/kexin type 9: a view beyond the canonical cholesterol-lowering impact. Am J Pathol.

[8] Zaid A, Roubtsova A, Essalmani R, Marcinkiewicz J, Chamberland A, Hamelin J (2008). Proprotein convertase subtilisin/kexin type 9 (PCSK9): hepatocyte-specific low-density lipoprotein receptor degradation and critical role in mouse liver regeneration. Hepatology.

[9] Sanz-Cuesta BE, Saver JL (2021). Lipid-lowering therapy and hemorrhagic stroke risk: comparative meta-analysis of statins and PCSK9 inhibitors. Stroke.

[10] Bergeron N, Phan BA, Ding Y, Fong A, Krauss RM (2015). Proprotein convertase subtilisin/kexin type 9 inhibition: a new therapeutic mechanism for reducing cardiovascular disease risk. Circulation.

[11] Dijk W, Cariou B (2019). Efficacy and safety of proprotein convertase subtilisin/kexin 9 inhibitors in people with diabetes and dyslipidaemia. Diabetes Obes Metab.

[12] Schmidt AF, Swerdlow DI, Holmes MV, Patel RS, Fairhurst-Hunter Z, Lyall DM (2017). PCSK9 genetic variants and risk of type 2 diabetes: a mendelian randomisation study. Lancet Diabetes Endocrinol.

[13] Lotta LA, Sharp SJ, Burgess S, Perry JRB, Stewart ID, Willems SM (2016). Association between low-density lipoprotein cholesterol-lowering genetic variants and risk of type 2 diabetes: a meta-analysis. JAMA.

[14] Khan SU, Talluri S, Riaz H, Rahman H, Nasir F, Bin Riaz I (2018). A Bayesian network meta-analysis of PCSK9 inhibitors, statins and ezetimibe with or without statins for cardiovascular outcomes. Eur J Prev Cardiol.

[15] Talasaz AH, Ho AJ, Bhatty F, Koenig RA, Dixon DL, Baker WL (2021). Meta-analysis of clinical outcomes of PCSK9 modulators in patients with established ASCVD. Pharmacotherapy.

[16] Hutton B, Salanti G, Caldwell DM, Chaimani A, Schmid CH, Cameron C (2015). The PRISMA extension statement for reporting of systematic reviews incorporating network meta-analyses of health care interventions: checklist and explanations. Ann Intern Med.

[17] Shinichi A (2014). Cochrane handbook for systematic reviews of interventions. Online Kensaku.

[18] Guyatt GH, Oxman AD, Vist GE, Kunz R, Falck-Ytter Y, Alonso-Coello P (2008). GRADE: an emerging consensus on rating quality of evidence and strength of recommendations. BMJ (Clinical research ed).

[19] Higgins JP, Thompson SG (2002). Quantifying heterogeneity in a meta-analysis. Stat Med.

[20] Egger M, Davey Smith G, Schneider M, Minder C (1997). Bias in meta-analysis detected by a simple, graphical test. BMJ (Clinical research ed).

[21] Ray KK, Wright RS, Kallend D, Koenig W, Leiter LA, Raal FJ (2020). Two phase 3 trials of Inclisiran in patients with elevated LDL cholesterol. N Engl J Med.

[22] Schwartz GG, Steg PG, Szarek M, Bhatt DL, Bittner VA, Diaz R (2018). Alirocumab and cardiovascular outcomes after acute coronary syndrome. N Engl J Med.

[23] Sabatine MS, Giugliano RP, Keech AC, Honarpour N, Wiviott SD, Murphy SA (2017). Evolocumab and clinical outcomes in patients with cardiovascular disease. N Engl J Med.

[24] Nicholls SJ, Puri R, Anderson T, Ballantyne CM, Cho L, Kastelein JJ (2016). Effect of Evolocumab on progression of coronary disease in statin-treated patients: the GLAGOV randomized clinical trial. JAMA.

[25] Robinson JG, Farnier M, Krempf M, Bergeron J, Luc G, Averna M (2015). Efficacy and safety of alirocumab in reducing lipids and cardiovascular events. N Engl J Med.

[26] Kereiakes DJ, Robinson JG, Cannon CP, Lorenzato C, Pordy R, Chaudhari U (2015). Efficacy and safety of the proprotein convertase subtilisin/kexin type 9 inhibitor alirocumab among high cardiovascular risk patients on maximally tolerated statin therapy: the ODYSSEY COMBO I study. Am Heart J.

[27] Cannon CP, Cariou B, Blom D, McKenney JM, Lorenzato C, Pordy R (2015). Efficacy and safety of alirocumab in high cardiovascular risk patients with inadequately controlled hypercholesterolaemia on maximally tolerated doses of statins: the ODYSSEY COMBO II randomized controlled trial. Eur Heart J.

[28] Raber L, Ueki Y, Otsuka T, Losdat S, Haner JD, Lonborg J (2022). Effect of alirocumab added to high-intensity statin therapy on coronary atherosclerosis in patients with acute myocardial infarction: the PACMAN-AMI randomized clinical trial. JAMA.

[29] Benjamin EJ, Virani SS, Callaway CW, Chamberlain AM, Chang AR, Cheng S (2018). Heart disease and stroke statistics-2018 update: a report from the American Heart Association. Circulation.

[30] Ridker PM, Tardif JC, Amarenco P, Duggan W, Glynn RJ, Jukema JW (2017). Lipid-reduction variability and antidrug-antibody formation with Bococizumab. N Engl J Med.

[31] Ferri N, Corsini A, Sirtori CR, Ruscica M (2017). Bococizumab for the treatment of hypercholesterolaemia. Expert Opin Biol Ther.

[32] Ma W, Guo X, Ma Y, Hu Z (2021). Meta-analysis of randomized clinical trials comparing PCSK9 monoclonal antibody versus ezetimibe/placebo in patients at high cardiovascular risk. Atherosclerosis.

[33] Geng Q, Li X, Sun Q, Wang Z (2021). Efficacy and safety of PCSK9 inhibition in cardiovascular disease: a meta-analysis of 45 randomized controlled trials. Cardiol J.

[34] Mu G, Xiang Q, Zhou S, Liu Z, Qi L, Jiang J (2020). Efficacy and safety of PCSK9 monoclonal antibodies in patients at high cardiovascular risk: an updated systematic review and meta-analysis of 32 randomized controlled trials. Adv Ther.

[35] Guedeney P, Sorrentino S, Giustino G, Chapelle C, Laporte S, Claessen BE (2021). Indirect comparison of the efficacy and safety of alirocumab and evolocumab: a systematic review and network meta-analysis. Eur Heart J Cardiovasc Pharmacother.

[36] Guedeney P, Giustino G, Sorrentino S, Claessen BE, Camaj A, Kalkman DN (2019). Efficacy and safety of alirocumab and evolocumab: a systematic review and meta-analysis of randomized controlled trials. Eur Heart J.

[37] Nicholls SJ, Kataoka Y, Nissen SE, Prati F, Windecker S, Puri R (2022). Effect of evolocumab on coronary plaque phenotype and burden in statin-treated patients following myocardial infarction. JACC Cardiovasc Imaging.

[38] Toth PP, Worthy G, Gandra SR, Sattar N, Bray S, Cheng LI (2017). Systematic review and network meta-analysis on the efficacy of evolocumab and other therapies for the management of lipid levels in hyperlipidemia. J Am Heart Assoc.

[39] Gupta M, Mancini GBJ, Wani RJ, Ahooja V, Bergeron J, Manjoo P (2022). Real-world insights into Evolocumab use in patients with hyperlipidemia: canadian analysis from the ZERBINI Study. CJC Open..

[40] Maliglowka M, Kosowski M, Hachula M, Cyrnek M, Buldak L, Basiak M (2022). Insight into the evolving role of PCSK9. Metabolites.

[41] Lorenzatti AJ, Monsalvo ML, López JAG, Wang H, Rosenson RS (2021). Effects of evolocumab in individuals with type 2 diabetes with and without atherogenic dyslipidemia: an analysis from BANTING and BERSON. Cardiovasc Diabetol.

[42] Fischer LT, Hochfellner DA, Knoll L, Pöttler T, Mader JK, Aberer F (2021). Real-world data on metabolic effects of PCSK9 inhibitors in a tertiary care center in patients with and without diabetes mellitus. Cardiovasc Diabetol.

[43] Reyes-Soffer G, Pavlyha M, Ngai C, Thomas T, Holleran S, Ramakrishnan R (2017). Effects of PCSK9 inhibition with alirocumab on lipoprotein metabolism in healthy humans. Circulation.

[44] Fitzgerald K, Frank-Kamenetsky M, Shulga-Morskaya S, Liebow A, Bettencourt BR, Sutherland JE (2014). Effect of an RNA interference drug on the synthesis of proprotein convertase subtilisin/kexin type 9 (PCSK9) and the concentration of serum LDL cholesterol in healthy volunteers: a randomised, single-blind, placebo-controlled, phase 1 trial. Lancet.

[45] Warden BA, Duell PB (2021). Inclisiran: a novel agent for lowering apolipoprotein b-containing lipoproteins. J Cardiovasc Pharmacol.

[46] Hao Q, Aertgeerts B, Guyatt G, Bekkering GE, Vandvik PO, Khan SU (2022). PCSK9 inhibitors and ezetimibe for the reduction of cardiovascular events: a clinical practice guideline with risk-stratified recommendations. BMJ.

[47] Kosmas CE, Muñoz Estrella A, Sourlas A, Silverio D, Hilario E, Montan PD (2018). Inclisiran: a new promising agent in the management of hypercholesterolemia. Diseases.

[48] Kam N, Perera K, Zomer E, Liew D, Ademi Z (2020). Inclisiran as adjunct lipid-lowering therapy for patients with cardiovascular disease: a cost-effectiveness analysis. Pharmacoeconomics.

