# Supplementary material for: PCSK9 inhibitors for secondary prevention in patients with cardiovascular diseases: a bayesian network meta-analysis
Source: Cardiovasc Diabetol. 2022 Jun 15;21:107. doi: 10.1186/s12933-022-01542-4 (PMC9202167; doi:10.1186/s12933-022-01542-4)
Supplement: Supplementary file 1 — Additional file 1: Table S1. Search Strategy. Figure S1. Study selection flowchart of randomized controlled trials. Figure S2. Risk of bias summary. Figure S3. Risk of bias graph. [file 12933_2022_1542_MOESM1_ESM.docx]

Supplemental e-material

| Table S1: Search Strategy  Figure S1: Study selection flowchart of randomized controlled trials |
| --- |
| Figure S2: Risk of bias summary |
| Figure S3: Risk of bias graph |

##### Table S1: Search Strategy:

| **OVID Medline (adapted for other databases)** | |
| --- | --- |
| 1 | exp PCSK9 Inhibitors/ |
| 2 | (Alirocumab or SAR236553 or Evolocumab or AMG 145 or Inclisiran). ab,kw,ti |
| 3 | #1 or #2 |
| 4 | exp hypercholesterolemia/ or exp familial hypercholesterolemia/ |
| 5 | (Hyperlipidemia or Dyslipidemia).ab,kw,ti |
| 6 | #4 or #5 |
| 7 | #3 and #6 |
| 8 | exp randomized controlled trial/ |
| 9 | (random* or blind* or placebo or trial*).ab,kw,ti. |
| 10 | 11 or 12 |
| 11 | exp human/ |
| 12 | 10 and 11 |
| 13 | 7 and 12 |

### **Figure S1: Study selection flowchart of randomized controlled trials**


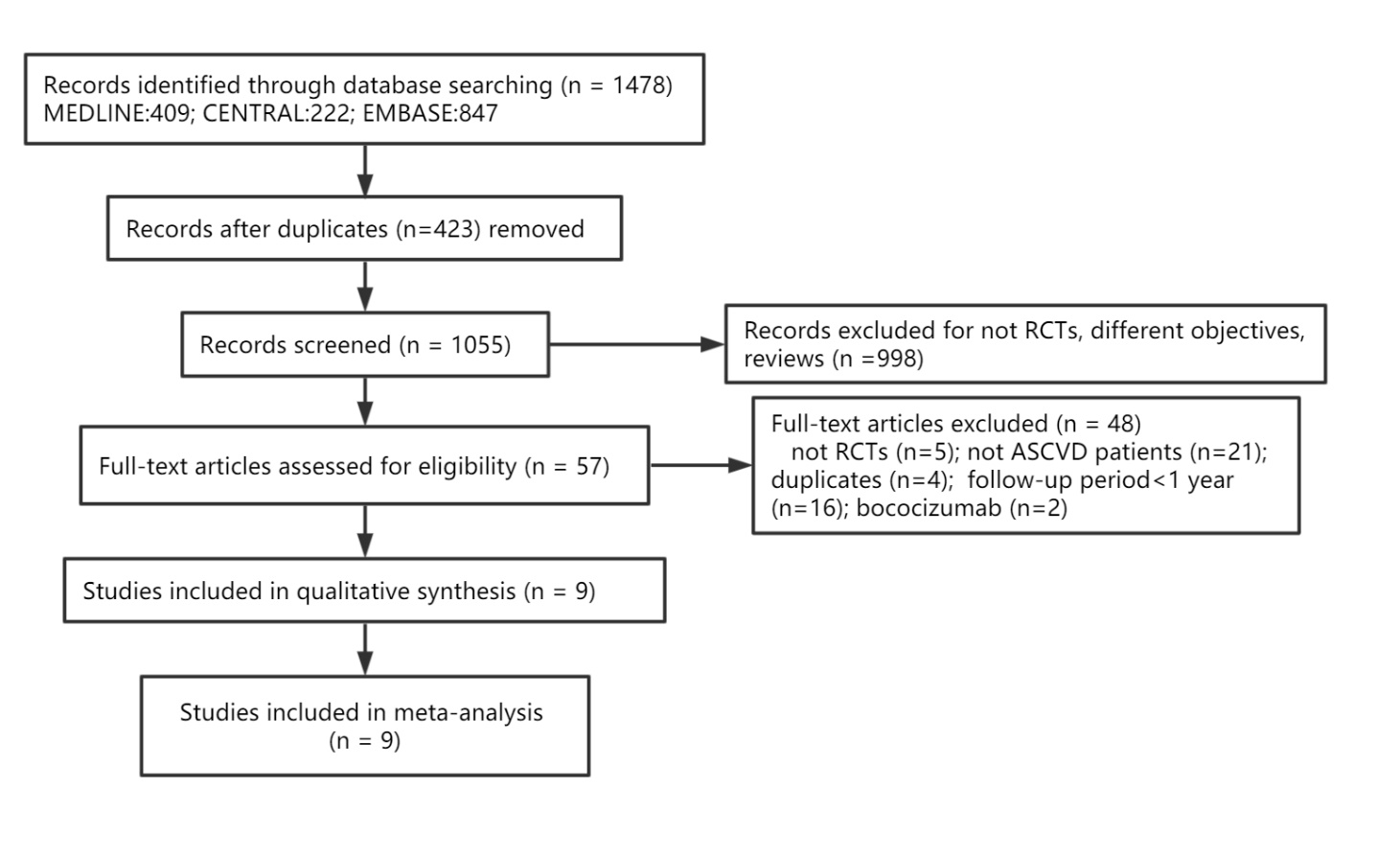


### **Figure S2: Risk of bias summary: review authors' judgements about each risk of bias item for each included study.**


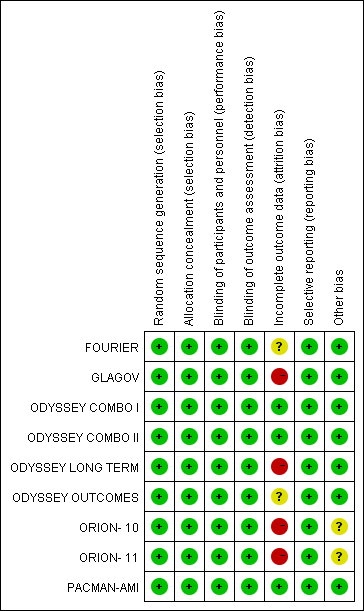


### **Figure S3: Risk of bias graph: review authors' judgements about each risk of bias item presented as percentages across all included studies**

**
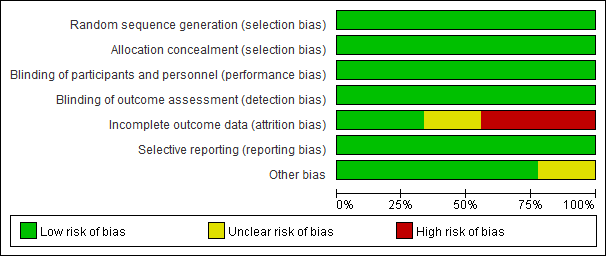
**
